# Preoperative nutrition in colorectal cancer surgery: mini review

**DOI:** 10.3389/fnut.2026.1922189

**Published:** 2026-08-28

**Authors:** Fernando Estrada-Moya, Salvador Ortiz-Gutiérrez, Martha Guevara-Cruz, Adriana Flores-López, Karla G. Hernández-Gómez, Luis E. González-Salazar, Laura A. Velázquez-Villegas, Rocío Guizar-Heredia, Laura Guevara-Pedraza, Aurora E. Serralde-Zúñiga

**Affiliations:** 1 Servicio de Nutriología Clínica, Instituto Nacional de Ciencias Médicas y Nutrición Salvador Zubirán, Ciudad de México, Mexico; 2 Sección de Estudios de Posgrado e Investigación, Instituto Politécnico Nacional “Escuela Superior de Medicina”, Ciudad de México, Mexico; 3 Departamiento de Fisiología de la Nutrición, Instituto Nacional de Ciencias Médicas y Nutrición Salvador Zubirán, Ciudad de México, Mexico; 4 Facultad de Ciencias de la Salud, Universidad Anáhuac Campus Sur, Ciudad de México, Mexico

**Keywords:** cancer-related sarcopenia, colorectal neoplasms, malnutrition, nutrition support, preoperative care

## Abstract

In colorectal cancer patients, malnutrition, sarcopenia, and even cachexia are highly prevalent (24–65%); those patients who undergo surgery with a compromised nutritional status tend to have more complications (OR: 1.84; 1.35–2.48). Therefore, proper nutritional management before surgery is essential. The principal therapeutic option for patients with colorectal cancer is surgery; in this way, these patients are vulnerable to a worse prognosis if a preoperative nutritional intervention is not considered. An individualized, well-designed nutrition care plan, as part of a multimodal therapeutic approach, that promotes adequate energy and protein intake to counteract catabolism and preserve muscle mass can help improve postoperative outcomes. Other strategies that may be useful for patients undergoing colorectal cancer surgery are immunonutrition and micronutrient supplementation. The combined use of certain immunonutrients, such as polyunsaturated fatty acids, branched-chain amino acids, arginine, glutamine, and nucleotides, has been suggested in some studies to help reduce surgery-related complications and infections, promote better wound healing, and shorten hospital stay after colorectal surgery. Certain micronutrients, such as vitamin C and zinc, can enhance the clinical effects of prior nutritional interventions and promote postoperative recovery. Nevertheless, limitations and mixed results in the current evidence preclude recommending these strategies in routine clinical practice. An individualized approach for each patient should be considered to improve surgical outcomes through nutrition.

## Introduction

Cancer is a disease caused by genetic alterations that lead to the uncontrolled growth and multiplication of cells (1). These cells also acquire invasive and metastatic properties and evade recognition and destruction by the immune system. Cancer can affect any organ or tissue in the human body (2). The main risk factors for cancer development are genetic mutations caused by errors in DNA replication, alongside aging; these two causes are classified as non-modifiable risk factors for cancer (3). Nevertheless, according to the World Health Organization (WHO), 35% of cancer cases are related to modifiable risk factors, with smoking and obesity being the major contributors (4).

Colorectal cancer is a heterogeneous disease that progresses slowly from a small benign polyp to a malignant tumor, which can take several years before symptom onset (5, 6). Data from the Global Cancer Observatory indicate that in 2020, colorectal cancer was the third most commonly diagnosed cancer worldwide (7). The incidence of colorectal cancer has increased in low- and middle-income nations. For instance, in México, the incidence of colorectal cancer was 7.8% for both sexes, and it was reported as the type of cancer with the greatest mortality, accounting for 8.6% of all deaths due to cancer recorded in 2022 (8). The risk of developing colon cancer increases with age, and it is estimated that the mean age of onset is 67 years. Despite this, around the world, it has been observed that even 10% of colorectal cancer cases occur in adults under 50 (9).

Despite most clinical guidelines for nutrition in surgery providing a framework for surgical cancer patients, some particularities regarding colorectal cancer remain unsettled, such as the use of specific nutrients or the benefit of immunonutrition. Therefore, this narrative mini review aims to explore the current evidence and present specific strategies to improve preoperative nutrition in patients undergoing colorectal cancer surgery. Relevant scientific evidence was identified through targeted searches; however, as a narrative review, no formal systematic search protocol was implemented.

## Colorectal cancer surgery

Treatment options for colorectal cancer may vary according to lesion localization along the colon or rectum, the stage at the time of diagnosis, the molecular profile of the tumor, and individual characteristics of the patient, such as age, functional status, comorbidities, social support, and personal preferences regarding treatment (6). Treatment modalities included surgery, chemotherapy, radiotherapy, cryotherapy, and immunotherapy, used in combination or in sequence. However, surgery remains the principal form of treatment for people living with this condition, particularly for stages I–III, representing the main way for curative treatment (5, 6, 9, 10).

Surgical treatment for patients who require partial or total colorectal resection includes both conventional open surgery and minimally invasive surgical procedures (5). Evidence shows that the laparoscopic surgical approach is comparable in efficacy and safety to the traditional open surgical approach (11, 12). The selection of one approach over the other depends on the specific tumor localization and on the physiological conditions of the patient; this ensures a personalized treatment to achieve the best possible outcomes. The most frequent surgical procedures are hemicolectomy, sigmoidectomy, low anterior resection, subtotal colectomy, and proctocolectomy, among others (13).

After the affected anatomical section is removed, the surgeon can re-establish continuity of the digestive tract through a primary anastomosis (14). Another option is to create a surgical intestinal ostomy to divert intestinal contents, thereby providing a protected environment that promotes wound healing (15). Intestinal diversion can be temporary or definitive, depending on the characteristics of the disease and prognosis. In those cases, with a high risk of dehiscence or anastomotic leakage, ostomy may provide a preventive strategy to improve surgical outcomes and reduce postoperative morbidity (16).

Finally, the decision between a primary anastomosis or an intestinal ostomy depends on multiple factors. In this way, the health team must consider the overall status of the patient, the integrity and perfusion of the involved tissues, the risk of infection, and the likelihood of other adverse events (17).

## Nutritional status and colorectal cancer

Nutrition status in people living with colorectal cancer is affected by the disease itself and by the treatment, leading to metabolic alterations, increased energy and protein requirements, and impaired nutrient absorption; also, the presence of symptoms such as anorexia, nausea, dysgeusia, among others (18). Consequently, all oncology patients have an increased risk of malnutrition; the prevalence of malnutrition has been reported to range from 20–70%, varying with age, disease stage, and treatments received. As well, about 10–20% of deaths among people living with cancer could be attributable to complications related to malnutrition, independently of the progression of the disease (19).

In people with cancer and malnutrition, the depletion of muscle proteins and the increased oxidation of essential amino acids, such as branched-chain amino acids (BCAAs), are common (20, 21). It has been observed that the concentration of some of these amino acids in circulation can affect the immune response and inflammatory status by stimulating lymphocyte proliferation, macrophage activation, and the production of proinflammatory cytokines (22).

Therefore, the reduction of BCAAs may lead to an increased inflammatory response and to compromised immunity. In certain situations, some amino acids may be considered essential even when they typically are not. This is the case for glutamine, whose levels drop during the trauma response or major surgery, compromising physiological processes that depend on this amino acid; metabolic stress has been reported to reduce muscle glutamine levels by up to 50% (23, 24).

Besides, the presence of sarcopenia, defined as low muscle strength, low muscle mass, and low physical performance (25, 26), is the most common nutritional issue present in people with cancer, with a great impact on disease prognosis. Sarcopenia is an independent predictor of poor quality of life, surgical complications, and lower survival (27).

The prevalence of low muscle mass is greater than 50% in newly diagnosed cancer patients, which importantly differs from the 15% among healthy individuals of similar age (28). As colorectal cancer progresses, the impact on nutritional status may manifest as cachexia, a multifactorial syndrome characterized by progressive loss of muscle mass, with or without fat mass loss, commonly associated with an inflammatory state (29).

Cancer-related cachexia represents a complex metabolic alteration that cannot be reversed entirely by conventional nutritional support (30). Therefore, the basic difference between cachexia and malnutrition lies in their potential for reversibility; when there is ongoing loss of weight and muscle mass despite adequate energy and protein intake, the likelihood of cancer-related cachexia is high (26). It is important to highlight that cancer-related cachexia can be identified in three consecutive stages: 1. Pre-cachexia (weight loss ≤5% and early metabolic changes); 2. Cachexia (weight loss >5%, or a body mass index (BMI) < 20 kg/m^2^ with ongoing weight loss >2%, or sarcopenia), and 3. Refractory cachexia (resistant catabolic state, low performance status, and life expectancy <3 months) (26). Not all patients go through the three stages; progression will depend on the type of cancer, the stage of the tumor, the presence of systemic inflammation, food intake, and the response to treatment (31). Cancer-related cachexia prevalence ranges from 50–80% (32); currently, there is no effective pharmacologic therapy to reverse cachexia completely (26).

## Impact of malnutrition on colorectal cancer surgery

In patients undergoing cancer surgery, malnutrition and systemic inflammation have been documented to increase the risk of postoperative complications (19). In the colorectal cancer surgery subset, approximately 24–65% of patients have malnutrition, increasing the risk of infections, morbidity, length of stay, and costs of medical care, as well as the risk of mortality (33, 34).

A compromised nutritional status, evidenced by malnutrition, sarcopenia, and/or cachexia, represents a risk factor for an adverse prognosis in any cancer surgery. Evidence shows that a poor nutritional status is independently associated with lower treatment tolerance, longer recovery after surgical procedures, and increased complications (35–42), as detailed in Table 1.

**Table 1:** Impact of compromised nutritional status on surgical outcomes.

Outcome	Findings	Value; 95% CI	*p*
Malnutrition			
Mortality	Overall mortality	HR: 2.04; 1.56–2.67 (40)	<0.001
	Hospital mortality	OR: 1.96; 1.73–2.23 (39)	<0.001
	Mortality in moderate malnutrition	HR: 1.44; 1.29–1.62 (41)	<0.001
	Mortality in severe malnutrition	HR: 1.79; 1.58–2.02 (41)	<0.001
Other complications	Overall complications	OR: 1.90; 1.41–2.55 (40)	<0.001
	Infections	OR: 2.38; 2.07–2.73 (39)	<0.001
	Surgical wound complications	OR: 3.32; 2.76–3.99 (39)	<0.001
	Postoperative bleeding	OR: 1.73; 1.44–2.07 (39)	<0.001
	Length of hospital stay	15.4 vs. 9.61 days (39)	<0.001
Economic burden	Hospitalization costs	$163,962 vs. $102,709 USD (39)	<0.001
Sarcopenia			
Mortality	Postoperative mortality	OR: 3.21; 2.01–5.11 (35)	<0.001
	30-day mortality	OR: 15.50; 2.0–120.0 (37)	0.009
	1-year mortality	OR: 16.2; 4.34–83.4 (38)	<0.01
Other complications	Overall complications	OR: 1.84; 1.35–2.48 (35)	<0.001
		OR: 3.42; 1.85–6.31 (36)	<0.001
	Severe complications	OR: 1.72; 1.10–2.69 (35)	0.02
	Anastomotic leakage	OR: 3.36; 1.12–10.12 (36)	0.03
	Surgical wound infection	OR: 3.02; 1.55–5.90 (36)	0.001
	Cardiopulmonary failure	OR: 2.92; 1.96–4.37 (35)	<0.001
	Infections	OR: 1.40; 1.12–1.76 (35)	0.003
	Length of hospital stay	MD: 0.77; 0.44–1.11 (35)	<0.001

CI, confidence interval; OR, odds ratio; HR, hazard ratio; USD, United States dollars; MD, mean difference.

Patients with malnutrition, hypoalbuminemia, and loss of appetite undergoing surgery have a greater risk of hospital mortality, which is five times higher compared to patients with a better nutritional status before surgery (39, 43, 44). Specifically, the presence of sarcopenia has an unfavorable effect on the postoperative evolution of colorectal surgery. Several meta-analyses have confirmed that reduced muscle mass and loss of muscle strength increase the risk of early mortality after surgery, from 30 days to 1 year. In the same way, sarcopenia leads to more chances of postoperative complications, such as infections, more adverse events related to the surgery, and cardiopulmonary failure (35, 37, 38).

On the other hand, compromised nutritional status may represent a significant burden on health systems due to increased resource use to manage malnutrition-related complications. Some data indicate that patients with malnutrition or sarcopenia undergoing colorectal surgery have longer hospital stays compared with well-nourished patients; this translates to an increase of 50% in hospital expenses for each admitted patient (38).

Nowadays, nutrition care is recognized as a human right; therefore, all people should have access to sufficient food and science-based medical nutrition therapy, including nutrition support and proper hydration (45). In patients with colorectal cancer, the nutritional intervention must focus on preventing malnutrition, especially on avoiding or reducing early loss of muscle mass (46). It is recommended to provide 25–30 kcal/kg/day and 1–1.5 g/kg/day of protein in patients with cancer (30, 47).

However, more than 80% of patients at advanced stages are unable to meet such requirements; the gap between nutrition prescribed and delivered remains a well-documented challenge in the hospital setting (48) but specific data in colorectal cancer surgery is still missing.

Currently, to increase the chances of treatment success, a multimodal approach is preferred, including the treatment for the underlying cancer, along with nutrition counseling, exercise-based interventions, anti-inflammatory agents, and symptom management, like pain, nausea, or dysphagia control, as well as mental health support, early palliative care, spiritual reinforcement, and always considering the preferences of the patients for the design of therapeutic schemes (32).

## Preoperative nutrition for colorectal cancer surgery

A good nutritional status is fundamental for all patients living with cancer. From those undergoing treatments such as surgery or chemotherapy to those in remission, proper nutritional support should be considered to improve clinical outcomes and quality of life. Even in the final stages of life, nutrition can positively impact the wellbeing of patients and should be considered part of palliative care (19). In this regard, nutrition support is indicated when patients can meet only 50–75% of their requirements for more than two weeks, or less than 50% for one week (47).

Nutrition risk screening is essential for the early identification of patients at risk of malnutrition who should be considered for a full nutritional assessment and an individualized nutrition care plan. The main interventions include dietary modifications, oral nutrition supplements (ONS), and enteral or parenteral nutrition as necessary (49).

**Figure 1:**
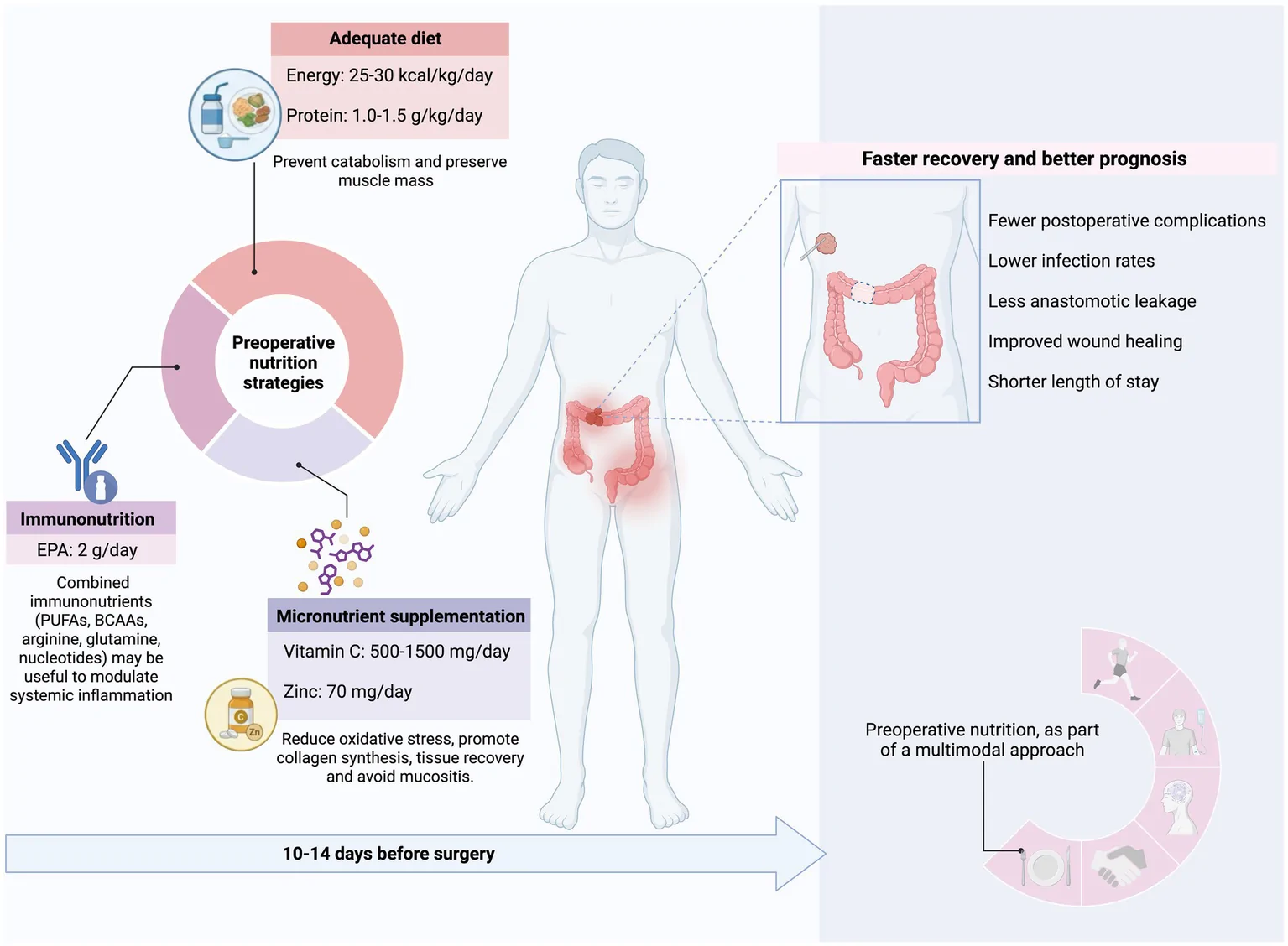
Preoperative nutrition strategies for colorectal cancer. The nutritional management of patients undergoing surgery for colorectal cancer must prioritize the adequate provision of energy (25–30 kcal/kg/day) and protein (1.0–1.5 g/kg/day) to counteract catabolism. Immunonutrition involving EPA (2 g/day) and micronutrient supplementation, including vitamin C (500–1,500 mg/day) and zinc (70 mg/day), may be considered as supplementary strategies; however, given the limited and mixed scientific evidence, a personalized assessment of their utility is advisable rather than routine implementation. Preoperative nutrition must be initiated 10–14 days prior to the intervention and as part of a multimodal therapeutic approach, to enhance surgical outcomes. BCAAs, branched-chain amino acids; EPA, eicosapentaenoic acid; PUFAs, n-3 polyunsaturated fatty acids. Created in BioRender. Ortiz, S. (2026) https://BioRender.com/96efbm7.

Preoperative nutrition strategies strongly promote the use of ONS, as they can provide approximately 500 kcal/day and, along with food intake, help meet energy and protein requirements (47). Evidence has also demonstrated that the use of ONS reduces postoperative complications and shortens length of stay (34). Various nutrition care algorithms suggest that patients undergoing elective surgery should receive preoperative nutrition interventions 10 to 14 days prior to surgery to improve nutritional status (50). Figure 1 summarizes the nutritional strategies for patients undergoing colorectal cancer surgery.

### Utility of immunonutrition

The use of nutrients such as n-3 polyunsaturated fatty acids (PUFAs), BCAAs, arginine, glutamine, and nucleotides, at supraphysiologic doses to modulate the immune response is called immunonutrition (51). In colorectal cancer, the use of formulas that combine immunonutrients, such as PUFAs, BCAAs, nucleotides, glutamine, and arginine, has been suggested to modulate the immune response, reducing postoperative infections, lowering the risk of dehiscence, anastomotic leakage, and shortening hospital stay (50, 52, 53).

A recent meta-analysis showed that enteral immunonutrition significantly reduced the risk of surgical wound infections (RR = 0.48, *p* = 0.0005) and decreased the length of stay after surgery by a mean of 2.37 days (*p* < 0.0001) in patients who underwent open colorectal surgery; such benefits were not observed for laparoscopic surgical approaches (54). It has also been recommended that preoperative immunonutrition be initiated at least seven days prior to elective surgery, ideally between 10–14 days before, by enteral route (50). Nevertheless, most evidence is based on the use of commercial nutrient blends rather than single nutrients; therefore, the observed effects result from synergistic interactions among components, complicating the isolation of clinical contributions attributable to any individual nutrient. Clinical outcomes associated with the use of immunonutrition should be interpreted within this context to prevent erroneous conclusions (54).

### Potential effect of immunonutrients

Evidence supports the effectiveness of certain nutrients that modulate the inflammatory response in the preoperative phase of cancer treatment; studies indicate that immunonutrition may improve surgical outcomes, enhance recovery, and improve quality of life as part of a well-planned, individualized nutrition intervention (55). However, the evidence regarding patients undergoing colorectal cancer surgery remains limited and inconclusive; therefore, the following statements synthesize evidence from mechanistic studies or draw conclusions from other patient groups. These should be regarded as plausible but must be considered in conjunction with the limitations of the current available evidence.

PUFAs are demonstrated to have utility in stabilizing the weight of patients with cancer with involuntary and progressive weight loss. These fatty acids can promote appetite, support muscle mass growth, and improve weight in patients with advanced cancer at high risk of malnutrition. Evidence also shows that 2 g/day of eicosapentaenoic acid is associated with reduced systemic inflammation and a reduced risk of postoperative infections (49). However, randomized controlled trials specifically evaluating PUFAs as isolated interventions in patients undergoing colorectal cancer surgery remain scarce, limiting the ability to determine their independent contribution to these clinical outcomes.

BCAAs, especially leucine, have a central role in muscle metabolism and the nutritional status of patients undergoing surgery for colorectal cancer. Leucine activates muscle protein synthesis and helps counteract the catabolic effects of surgical stress (56). The use of BCAA-enriched formulations has been shown to increase muscle protein synthesis and to promote glutamine production, which is essential for immune response and overall recovery after surgery (57); although mechanistic explanations may be plausible for these effects, there is lack of randomized controlled clinical trials to support them in the clinical setting.

Glutamine is an amino acid that plays a fundamental role in the metabolism of rapidly turning-over cells, particularly in enterocytes and colonocytes, acts as a precursor in protein synthesis, and is also necessary for the preservation of the gut mucosa. Immune cells use glutamine as their primary energy source; therefore, this amino acid contributes to a proper immune response; however, clinical evidence for glutamine use has shown mixed results, limiting reliable recommendations (24, 58, 59).

Meanwhile, arginine is needed in the wound-healing process due to its essential role in collagen synthesis and cell proliferation, making it crucial for wound recovery after colorectal surgery (60). Scientific evidence indicates that preoperative supplementation with arginine may improve immune response by increasing immunoglobulin and CD4 + T lymphocyte levels, which is associated with fewer surgical wound infections and less anastomotic leakage, as well as a shorter hospital stay after colorectal surgery (61). Nevertheless, in clinical studies, arginine is frequently provided through nutrient blends, making it difficult to establish independent therapeutic effects.

Lastly, it is proposed that specific immune-response-modulating nutrients be integrated into a nutrition care plan rather than used in isolation, with the main goal of potentiating their clinical effect. In this way, the combined use of immunonutrients in special formulations, along with appropriate provision of energy and protein, may enable an effective modulation of systemic inflammation, preservation of muscle mass, promotion of wound healing, and postoperative recovery (46). However, as discussed, the clinical evidence supporting specific outcomes is limited; consequently, conclusions are frequently drawn from theoretical physiological reasoning. Therefore, caution is warranted regarding declared clinical efficacy, particularly when observations originate from mechanistic studies or preclinical models.

### Micronutrient supplementation

To improve the conditions for patients undergoing colorectal cancer surgery, it has been suggested that, in addition to immunonutrition, preoperative use of specific micronutrients, such as vitamin C and zinc, should be considered (62, 63). Currently, there is insufficient high-quality evidence to demonstrate a clinical benefit of using such micronutrients in colorectal cancer. Therefore, none of the guidelines for the nutritional management of these patients state that micronutrient supplementation should be part of routine intervention (47).

However, some studies support positive physiological effects on metabolic and immune response. Additionally, micronutrient supplementation may be useful for patients with subclinical or clinical deficiencies in these micronutrients, the prevalence of which may be high among people living with cancer (63, 64).

#### Vitamin C

Vitamin C, or ascorbic acid, is essential for collagen synthesis, antioxidant activity, and tissue healing processes. In patients with cancer, a decrease in vitamin C concentrations has been observed, which may be related to reduced dietary intake, systemic inflammation due to disease, or oncologic treatments (65). In the immediate postoperative period, therapeutic supplementation with vitamin C has been associated with reduced patient-reported pain, which translates into reduced morphine requirements (66).

It is common for plasma vitamin C concentrations to decrease after surgical procedures due to increased acute oxidative stress. To help normalize concentrations, stable patients may receive vitamin C doses ranging from 500 mg/day to 1,500 mg/day (67). Furthermore, it has been shown that parenteral administration of vitamin C at 500 mg/day significantly reduces oxidative stress biomarkers in patients after colorectal surgery (68). Regarding tissue healing, the most robust results have been observed when vitamin C is supplemented in patients with pressure ulcers; however, evidence for its usefulness in surgical wound healing is scarce (67).

#### Zinc

Zinc is a trace mineral essential for humans, primarily as a cofactor in numerous enzymes and transcription factors; it participates in metalloproteinases, promotes DNA synthesis, and contributes to the immune response, thereby making fundamental contributions to wound healing (69). As with other trace elements, zinc deficiency is common among patients with cancer, which could worsen after surgical procedures, especially in those patients undergoing gastrointestinal surgery (70).

Clinical trials across different cancer types have indicated a potential therapeutic role for zinc. For instance, oral zinc sulfate supplementation at 25 mg three times a day has significantly reduced the frequency and severity of radiotherapy-induced mucositis and dermatitis in patients with head and neck cancer. In this context, a recent meta-analysis concluded that oral zinc administration at doses of 150 mg/day or higher is effective in preventing chemotherapy- or radiotherapy-induced oral mucositis (71, 72).

In colorectal cancer, a randomized clinical trial showed that administering 70 mg/day of zinc during active chemotherapy increased the biological activity of the enzyme superoxide dismutase and maintained vitamin E concentrations. These findings suggest improved antioxidant mechanisms and potential cellular protection against chemotherapy-induced toxicity and metabolic damage (73). It is important to consider that the amount of zinc may vary depending on the specific pharmaceutical salt used (74).

In clinical practice, there is no consensus on specific doses of preoperative micronutrient supplementation for people with cancer. Nevertheless, the most common therapeutic regimens used in clinical trials in the preoperative phase are as follows: Vitamin C (orally), 500–1,500 mg/day in patients undergoing surgery (65, 66); Zinc (orally), 70 mg/day in patients undergoing surgery and during adjuvant chemotherapy or radiotherapy (70, 75).

## Future directions

Medical Nutrition therapy for patients with colorectal cancer represents a challenge. Preserving or correcting nutritional status should be the primary objective of any nutritional intervention in this group before initiating cancer therapeutic approaches, such as surgery (47). Colorectal cancer itself and its management may lead to metabolic impairment, malnutrition, sarcopenia, or even cachexia, which negatively impact postoperative outcomes (19). Accordingly, preoperative nutrition for patients undergoing colorectal cancer surgery should be well established, with clear therapeutic plans and defined goals for assessing intervention success based on the nutritional status of each patient (33).

Although strategies, such as immunonutrition and micronutrient supplementation, have shown promising results for improving postoperative outcomes, the current evidence remains inconclusive regarding their true benefit when compared with standard nutritional care in gastrointestinal cancer surgery (76). The heterogeneity of study designs, the inconsistent findings, and the lack of specific evidence of the studies, the mixed results, and the lack of specific evidence for colorectal cancer surgery limit the ability to make a solid recommendation for the use of such interventions in this group of patients.

The gap in the evidence results from methodological difficulties inherent to colorectal cancer research that make high-quality studies in this heterogeneous group complex. Key factors that may challenge proper research include, among others, the type and stage of tumor, the localization along the colon or rectum, the selected surgical approach, whether open or laparoscopic, the existence of adjuvant therapies, the age at cancer onset and diagnosis, as well as the nutritional, metabolic, and inflammatory status before surgery (77).

Consequently, larger studies are needed to support specific nutritional interventions for colorectal cancer surgery (52). Future research should address these methodological barriers by stratifying clinical trials to account for disease diversity; studies should also elucidate whether the use of certain nutrients at specific doses reflects a direct therapeutic effect or merely corrects underlying deficiencies. Clarifying these aspects will be fundamental to establishing evidence-based recommendations.

## Limitations

The findings discussed here are based on a narrative review of the current scientific evidence on the subject. Still, it is important to note that the absence of a systematic search protocol may lead to biased selection of the studies discussed. Most evidence on immunonutrition and micronutrient supplementation comes from diverse cancer patient populations, limited clinical trials, or mechanistic investigations, which restricts the applicability of these findings to colorectal cancer patients. Accordingly, the recommendations presented here should be considered in light of the scope of this type of study. Further research is needed to establish more precise evidence-based statements.

## Conclusion

Any patient with compromised nutritional status who undergoes surgery without a proper nutrition care plan is highly likely to experience surgery-related complications, longer hospital stays, increased medical costs, and an overall worse prognosis, regardless of the surgical procedure. Therefore, when managing patients undergoing colorectal cancer surgery, clinicians should prioritize timely nutrition risk screening, preoperative nutrition interventions at least 10 days before surgery, and appropriate provision of energy and protein. Preoperative nutrition, as part of a multimodal approach for colorectal cancer, special formulations with combined immunonutrients, and strategic supplementation of certain micronutrients, may also be favorable for improving clinical outcomes after colorectal surgery. However, nutrition therapy should always be individualized to the disease characteristics, patient condition, and anticipated surgical intervention to achieve the best clinical outcomes after the procedure.

## References

[1] Vaghari-Tabari M Ferns GA Qujeq D Andevari AN Sabahi Z Moein S. Signaling, metabolism, and cancer: an important relationship for therapeutic intervention. J Cell Physiol. (2021) 236:5512–32. doi: 10.1002/jcp.30276, 33580511

[2] Hanahan D Weinberg RA. Hallmarks of cancer: the next generation. Cell. (2011) 144:646–74. doi: 10.1016/j.cell.2011.02.013, 21376230

[3] Lewandowska A Rudzki M Rudzki S Lewandowski T Laskowska B. Environmental risk factors for cancer—review paper. Ann Agric Environ Med. (2019) 26:1–7. doi: 10.26444/aaem/94299, 30922021

[4] Yıldırım-Kahrıman S. Non-intrinsic cancer risk factors. Exp Oncol. (2023) 43:290–7. doi: 10.32471/exp-oncology.2312-8852.vol-43-no-4.16804 34967549

[5] Shinji S Yamada T Matsuda A Sonoda H Ohta R Iwai T . Recent advances in the treatment of colorectal Cancer: a review. J Nippon Med Sch. (2022) 89:246–54. doi: 10.1272/jnms.JNMS.2022_89-310, 35082204

[6] Adebayo AS Agbaje K Adesina SK Olajubutu O. Colorectal cancer: disease process, current treatment options, and future perspectives. Pharmaceutics. (2023) 15:2620. doi: 10.3390/pharmaceutics15112620, 38004598 PMC10674471

[7] Morgan E Arnold M Gini A Lorenzoni V Cabasag CJ Laversanne M . Global burden of colorectal cancer in 2020 and 2040: incidence and mortality estimates from GLOBOCAN. Gut. (2023) 72:338–44. doi: 10.1136/gutjnl-2022-327736, 36604116

[8] Bray F Laversanne M Sung H Ferlay J Siegel RL Soerjomataram I . Global cancer statistics 2022: GLOBOCAN estimates of incidence and mortality worldwide for 36 cancers in 185 countries. CA Cancer J Clin. (2024) 74:229–63. doi: 10.3322/caac.21834, 38572751

[9] Eng C Yoshino T Ruíz-García E Mostafa N Cann CG O’Brian B . Colorectal cancer. Lancet. (2024) 404:294–310. doi: 10.1016/S0140-6736(24)00360-X, 38909621

[10] Wagle NS Nogueira L Devasia TP Mariotto AB Yabroff KR Islami F . Cancer treatment and survivorship statistics, 2025. CA Cancer J Clin. (2025) 75:308–40. doi: 10.3322/caac.70011, 40445120 PMC12223361

[11] Inomata M Yasuda K Shiraishi N Kitano S. Clinical evidences of laparoscopic versus open surgery for colorectal cancer. Jpn J Clin Oncol. (2009) 39:471–7. doi: 10.1093/jjco/hyp063, 19556338

[12] Bonjer HJ Deijen CL Abis GA Cuesta MA Van Der Pas MHGM De Lange-de Klerk ESM . A randomized trial of laparoscopic versus open surgery for rectal cancer. N Engl J Med. (2015) 372:1324–32. doi: 10.1056/NEJMoa1414882, 25830422

[13] Ng SC McCombie A Frizelle F Eglinton T. Influence of the type of anatomic resection on anastomotic leak after surgery for colon cancer. ANZ J Surg. (2024) 94:424–8. doi: 10.1111/ans.18782, 37990637

[14] Parker JM Feldmann TF Cologne KG. Advances in laparoscopic colorectal surgery. Surg Clin N Am. (2017) 97:547–60. doi: 10.1016/j.suc.2017.01.005, 28501246

[15] Dawes AJ Gahagan JV. Stoma complications. Clin Colon Rectal Surg. (2024) 37:387–97. doi: 10.1055/s-0043-1777453, 39399130 PMC11466528

[16] Werner JM Kupke P Ertl M Opitz S Schlitt HJ Hornung M. Timing of closure of a protective loop-ileostomy can be crucial for restoration of a functional digestion. Front Surg. (2022) 9:821509. doi: 10.3389/fsurg.2022.821509, 35419403 PMC8999839

[17] Ekdal DC Demirbaş BT Altunsu S Yeğen ŞC. Outcomes of colostomy versus primary anastomosis in left-sided obstructive colon cancer. Surg Pract. (2026). 1–7. doi: 10.1111/1744-1633.70069

[18] Viana ECRDM Oliveira IDS Rechinelli AB Marques IL Souza VFD Spexoto MCB . Malnutrition and nutrition impact symptoms (NIS) in surgical patients with cancer. PLoS One. (2020) 15:e0241305. doi: 10.1371/journal.pone.0241305, 33320857 PMC7737886

[19] Arends J Baracos V Bertz H Bozzetti F Calder PC Deutz NEP . ESPEN expert group recommendations for action against cancer-related malnutrition. Clin Nutr. (2017) 36:1187–96. doi: 10.1016/j.clnu.2017.06.017, 28689670

[20] Wei Z Liu X Cheng C Yu W Yi P. Metabolism of amino acids in cancer. Front Cell Dev Biol. (2021) 8:603837. doi: 10.3389/fcell.2020.603837, 33511116 PMC7835483

[21] Lai H-S Lee J-C Lee P-H Wang S-T Chen W-J. Plasma free amino acid profile in cancer patients. Semin Cancer Biol. (2005) 15:267–76. doi: 10.1016/j.semcancer.2005.04.003, 15894488

[22] Li P Yin Y-L Li D Woo Kim S Wu G. Amino acids and immune function. Br J Nutr. (2007) 98:237–52. doi: 10.1017/S000711450769936X, 17403271

[23] Xiong K Li G Zhang Y Bao T Li P Yang X . Effects of glutamine on plasma protein and inflammation in postoperative patients with colorectal cancer: a meta-analysis of randomized controlled trials. Int J Color Dis. (2023) 38:212. doi: 10.1007/s00384-023-04504-8, 37566134 PMC10421765

[24] Vijey AM Meha RJ. Enteral/oral glutamine supplementation in patients following surgery and accidental injury. Asian J Pharm Clin Res. (2017) 10:477. doi: 10.22159/ajpcr.2017.v10i3.16569

[25] Cruz-Jentoft AJ Bahat G Bauer J Boirie Y Bruyère O Cederholm T . Sarcopenia: revised European consensus on definition and diagnosis. Age Ageing. (2019) 48:16–31. doi: 10.1093/ageing/afy169, 30312372 PMC6322506

[26] Fearon K Strasser F Anker SD Bosaeus I Bruera E Fainsinger RL . Definition and classification of cancer cachexia: an international consensus. Lancet Oncol. (2011) 12:489–95. doi: 10.1016/S1470-2045(10)70218-7, 21296615

[27] Prado CM Purcell SA Laviano A. Nutrition interventions to treat low muscle mass in cancer. J Cachexia Sarcopenia Muscle. (2020) 11:366–80. doi: 10.1002/jcsm.12525, 31916411 PMC7113510

[28] Von Haehling S Morley JE Anker SD. An overview of sarcopenia: facts and numbers on prevalence and clinical impact. J Cachexia Sarcopenia Muscle. (2010) 1:129–33. doi: 10.1007/s13539-010-0014-2, 21475695 PMC3060646

[29] Ni J Zhang L. Cancer cachexia: definition, staging, and emerging treatments. CMAR. (2020) 12:5597–605. doi: 10.2147/CMAR.S261585, 32753972 PMC7358070

[30] Fioretti F Von Haehling S Coats AJS Butler J Del Fabbro E Skipworth RJE . Cachexia and wasting in chronic illness: regulatory and clinical trial update. J Cachexia Sarcopenia Muscle. (2025) 16:e70128. doi: 10.1002/jcsm.70128, 41305948 PMC12658287

[31] Roeland EJ Bohlke K Baracos VE Bruera E Del Fabbro E Dixon S . Management of cancer cachexia: ASCO guideline. JCO. (2020) 38:2438–53. doi: 10.1200/JCO.20.00611, 32432946

[32] Reid J Blair C Dempster M McKeaveney C Slee A Fitzsimons D. Multimodal interventions for cachexia management. Cochrane Database Syst Rev. (2025) 3:CD015749. doi: 10.1002/14651858.CD015749.pub2, 40130780 PMC11934851

[33] Wischmeyer PE Carli F Evans DC Guilbert S Kozar R Pryor A . American Society for Enhanced Recovery and Perioperative Quality Initiative Joint Consensus Statement on nutrition screening and therapy within a surgical enhanced recovery pathway. Anesth Analg. (2018) 126:1883–95. doi: 10.1213/ANE.0000000000002743, 29369092

[34] Gupta A Gupta E Hilsden R Hawel JD Elnahas AI Schlachta CM . Preoperative malnutrition in patients with colorectal cancer. CJS. (2021) 64:E621–9. doi: 10.1503/cjs.016820, 34824150 PMC8628841

[35] Trejo-Avila M Bozada-Gutiérrez K Valenzuela-Salazar C Herrera-Esquivel J Moreno-Portillo M. Sarcopenia predicts worse postoperative outcomes and decreased survival rates in patients with colorectal cancer: a systematic review and meta-analysis. Int J Color Dis. (2021) 36:1077–96. doi: 10.1007/s00384-021-03839-4, 33481108

[36] Su S Lin Z Cai Z Huang L Xiao Y Yang F . Preoperative CT-derived sarcopenia as a predictor of postoperative complications in patients undergoing laparoscopic radical resection for non-metastatic colorectal cancer: a retrospective study. Int J Color Dis. (2025) 40:140. doi: 10.1007/s00384-025-04932-8, 40517183 PMC12167240

[37] Reisinger KW Van Vugt JLA Tegels JJW Snijders C Hulsewé KWE Hoofwijk AGM . Functional compromise reflected by sarcopenia, frailty, and nutritional depletion predicts adverse postoperative outcome after colorectal cancer surgery. Ann Surg. (2015) 261:345–52. doi: 10.1097/SLA.0000000000000628, 24651133

[38] Richards SJG Senadeera SC Frizelle FA. Sarcopenia, as assessed by psoas cross-sectional area, is predictive of adverse postoperative outcomes in patients undergoing colorectal cancer surgery. Dis Colon Rectum. (2020) 63:807–15. doi: 10.1097/DCR.0000000000001633, 32149784

[39] Lee DU Fan GH Hastie DJ Addonizio EA Suh J Prakasam VN . The clinical impact of malnutrition on the postoperative outcomes of patients undergoing colorectal resection surgery for colon or rectal cancer: propensity score matched analysis of 2011–2017 US hospitals. Surg Oncol. (2021) 38:101587. doi: 10.1016/j.suronc.2021.101587, 33915485

[40] Zhang J Quan Y Wang X Wei X Shen X Li X . Global epidemiological characteristics of malnutrition in cancer patients: a comprehensive meta-analysis and systematic review. BMC Cancer. (2025) 25:1191. doi: 10.1186/s12885-025-14558-2, 40684092 PMC12275410

[41] Yin L Chong F Huo Z Li N Liu J Xu H. GLIM-defined malnutrition and overall survival in cancer patients: a meta-analysis. J Parenter Enter Nutr. (2023) 47:207–19. doi: 10.1002/jpen.2463, 36371641 PMC10107432

[42] Pimiento JM Evans DC Tyler R Barrocas A Hernandez B Araujo-Torres K . Value of nutrition support therapy in patients with gastrointestinal malignancies: a narrative review and health economic analysis of impact on clinical outcomes in the United States. J Gastrointest Oncol. (2021) 12:864–73. doi: 10.21037/jgo-20-326, 34012673 PMC8107619

[43] Trujillo EB Kadakia KC Thomson C Zhang FF Livinski A Pollard K . Malnutrition risk screening in adult oncology outpatients: an ASPEN systematic review and clinical recommendations. J Parenter Enter Nutr. (2024) 48:874–94. doi: 10.1002/jpen.2688, 39412097

[44] Muscaritoli M Modena A Valerio M Marchetti P Magarotto R Quadrini S . The impact of Nutritional status at first medical oncology visit on clinical outcomes: the NUTRIONCO study. Cancer. (2023) 15:3206. doi: 10.3390/cancers15123206, 37370816 PMC10296396

[45] Cardenas D Correia MITD Ochoa JB Hardy G Rodriguez-Ventimilla D Bermúdez CE . Clinical nutrition and human rights. An international position paper. Clin Nutr. (2021) 40:4029–36. doi: 10.1016/j.clnu.2021.02.039 34023070

[46] Tanaka K Nakamura S Narimatsu H. Nutritional approach to cancer Cachexia: a proposal for dietitians. Nutrients. (2022) 14:345. doi: 10.3390/nu14020345, 35057531 PMC8779386

[47] Muscaritoli M Arends J Bachmann P Baracos V Barthelemy N Bertz H . ESPEN practical guideline: Clinical Nutrition in cancer. Clin Nutr. (2021) 40:2898–913. doi: 10.1016/j.clnu.2021.02.005, 33946039

[48] Ben Othman R Cherni A Dergaa I Ceylan Hİ Ceylan NB Stefanica V . High prevalence of malnutrition and sarcopenia with inadequate nutritional support in intensive care unit patients: a prospective observational study of clinical outcomes. Nutrients. (2026) 18:883. doi: 10.3390/nu18060883, 41901057 PMC13029265

[49] August DA Huhmann MB. A.S.P.E.N. Clinical guidelines: nutrition support therapy during adult anticancer treatment and in hematopoietic cell transplantation. J Parenter Enter Nutr. (2009) 33:472–500. doi: 10.1177/0148607109341804, 19713551

[50] Weimann A Bezmarevic M Braga M Correia MITD Funk-Debleds P Gianotti L . ESPEN guideline on clinical nutrition in surgery—update 2025. Clin Nutr. (2025) 53:222–61. doi: 10.1016/j.clnu.2025.08.029, 40957230

[51] Yeh DD. Fueling recovery: evidence-based ICU nutrition and immunonutrition strategies in 2026. Trauma Surg Acute Care Open. (2026) 11:e002284. doi: 10.1136/tsaco-2026-002284, 42094750 PMC13141161

[52] Yu K Zheng X Wang G Liu M Li Y Yu P . Immunonutrition vs. standard nutrition for cancer patients: a systematic review and meta-analysis (part 1). J Parenter Enter Nutr. (2020) 44:742–67. doi: 10.1002/jpen.1736, 31709584

[53] Gustafsson UO Scott MJ Hubner M Nygren J Demartines N Francis N . Guidelines for perioperative care in elective colorectal surgery: enhanced recovery after surgery (ERAS®) society recommendations: 2018. World J Surg. (2019) 43:659–95. doi: 10.1007/s00268-018-4844-y, 30426190

[54] Wong CS Zaman S Siddiraju K Sellvaraj A Ghattas T Tryliskyy Y. Effects of enteral immunonutrition in laparoscopic versus open resections in colorectal cancer surgery: a meta-analysis of randomised controlled trials. Eur J Surg Oncol. (2025) 51:109488. doi: 10.1016/j.ejso.2024.109488, 39708458

[55] García-Malpartida K Aragón-Valera C Botella-Romero F Ocón-Bretón MJ López-Gómez JJ. Effects of immunonutrition on cancer patients undergoing surgery: a scoping review. Nutrients. (2023) 15:1776. doi: 10.3390/nu15071776, 37049616 PMC10096769

[56] Soares JDP Howell SL Teixeira FJ Pimentel GD. Dietary amino acids and immunonutrition supplementation in cancer-induced skeletal muscle mass depletion: a mini-review. CPD. (2020) 26:970–8. doi: 10.2174/1381612826666200218100420, 32067606

[57] Choudry HA Pan M Karinch AM Souba WW. Branched-chain amino acid-enriched nutritional support in surgical and cancer patients. J Nutr. (2006) 136:314S–8S. doi: 10.1093/jn/136.1.314S, 16365105

[58] Tang G Pi F Qiu Y-H Wei Z-Q. Postoperative parenteral glutamine supplementation improves the short-term outcomes in patients undergoing colorectal cancer surgery: a propensity score matching study. Front Nutr. (2023) 10:1040893. doi: 10.3389/fnut.2023.1040893, 37006941 PMC10060866

[59] Spittler A Sautner T Gornikiewicz A Manhart N Oehler R Bergmann M . Postoperative glycyl-glutamine infusion reduces immunosuppression: partial prevention of the surgery induced decrease in HLA-DR expression on monocytes. Clin Nutr. (2001) 20:37–42. doi: 10.1054/clnu.2000.0153, 11161542

[60] Witte MB Vogt N Stuelten C Gotoh T Mori M Becker HD. Arginase acts as an alternative pathway of L-arginine metabolism in experimental colon anastomosis. J Gastrointest Surg. (2003) 7:378–85. doi: 10.1016/S1091-255X(02)00431-6, 12654563

[61] Ouyang Z Chen P Zhang M Wu S Qin Z Zhou L. Arginine on immune function and post-operative obstructions in colorectal cancer patients: a meta-analysis. BMC Cancer. (2024) 24:1089. doi: 10.1186/s12885-024-12858-7, 39223466 PMC11370068

[62] Liu M-Y Tang H-C Hu S-H Yang H-L Chang S-J. Influence of preoperative peripheral parenteral nutrition with micronutrients after colorectal cancer patients. Biomed Res Int. (2015) 2015:1–6. doi: 10.1155/2015/535431, 26000296 PMC4426776

[63] Gröber U Holzhauer P Kisters K Holick M Adamietz I. Micronutrients in oncological intervention. Nutrients. (2016) 8:163. doi: 10.3390/nu8030163, 26985904 PMC4808891

[64] Zhu W Liu C Yao K Zhang X Huang J Cheng M . Micronutrient deficiency detected by liquid chromatography-tandem mass spectrometry and its association with clinical outcome of cancer patients—a prospective cohort study. BMC Med. (2025) 23:539. doi: 10.1186/s12916-025-04370-x, 41063161 PMC12506358

[65] Abiri B Vafa M. Vitamin C and cancer: the role of vitamin C in disease progression and quality of life in cancer patients. Nutr Cancer. (2021) 73:1282–92. doi: 10.1080/01635581.2020.1795692, 32691657

[66] Suter M Bollen Pinto B Belletti A Putzu A. Efficacy and safety of perioperative vitamin C in patients undergoing noncardiac surgery: a systematic review and meta-analysis of randomised trials. Br J Anaesth. (2022) 128:664–78. doi: 10.1016/j.bja.2021.11.039, 35090721

[67] Bechara N Flood VM Gunton JE. A systematic review on the role of vitamin C in tissue healing. Antioxidants. (2022) 11:1605. doi: 10.3390/antiox11081605, 36009324 PMC9405326

[68] Fukushima R Yamazaki E. Vitamin C requirement in surgical patients. Curr Opin Clin Nutr Metab Care. (2010) 13:669–76. doi: 10.1097/MCO.0b013e32833e05bc, 20689415

[69] Duan M Li T Liu B Yin S Zang J Lv C . Zinc nutrition and dietary zinc supplements. Crit Rev Food Sci Nutr. (2023) 63:1277–92. doi: 10.1080/10408398.2021.1963664, 34382897

[70] Lansdown ABG Mirastschijski U Stubbs N Scanlon E Ågren MS. Zinc in wound healing: theoretical, experimental, and clinical aspects. Wound Repair Regen. (2007) 15:2–16. doi: 10.1111/j.1524-475X.2006.00179.x, 17244314

[71] Lin L-C Que J Lin L-K Lin F-C. Zinc supplementation to improve mucositis and dermatitis in patients after radiotherapy for head-and-neck cancers: a double-blind, randomized study. Int J Radiat Oncol Biol Phys. (2006) 65:745–50. doi: 10.1016/j.ijrobp.2006.01.015, 16751063

[72] Wang J. Time-dependent efficacy of zinc supplements in preventing oral mucositis after chemoradiotherapy: a meta-analysis. Support Care Cancer. (2026) 34:358. doi: 10.1007/s00520-026-10572-7, 41872524

[73] Ribeiro SMDF Braga CBM Peria FM Domenici FA Martinez EZ Feres O . Effect of zinc supplementation on antioxidant defenses and oxidative stress markers in patients undergoing chemotherapy for colorectal cancer: a placebo-controlled, prospective randomized trial. Biol Trace Elem Res. (2016) 169:8–16. doi: 10.1007/s12011-015-0396-2, 26066525

[74] Devarshi PP Mao Q Grant RW Hazels Mitmesser S. Comparative absorption and bioavailability of various chemical forms of zinc in humans: a narrative review. Nutrients. (2024) 16:4269. doi: 10.3390/nu16244269, 39770891 PMC11677333

[75] Takishita C Nagakawa Y Osakabe H Nakagawa N Mitsuka Y Mazaki J . Significance of zinc replacement therapy after pancreaticoduodenectomy. Anticancer Res. (2022) 42:5833–7. doi: 10.21873/anticanres.16091, 36456161

[76] Sakhri S Othman RB Jerbi C Ceylan Hİ Naija L Zemni I . Effect of perioperative supplementation with arginine and Omega-3 on postoperative complications in patients undergoing gastrointestinal cancer surgery: a pilot open-label randomized controlled trial. Nutrients. (2026) 18:651. doi: 10.3390/nu18040651, 41754168 PMC12942923

[77] Qi G-X Zhao R-X Gao C Ma Z-Y Wang S Xu J. Recent advances and challenges in colorectal cancer: from molecular research to treatment. World J Gastroenterol. (2025) 31:1–30. doi: 10.3748/wjg.v31.i21.106964, 40538516 PMC12175868

